# Impaired motor skill learning and altered seizure susceptibility in mice with loss or gain of function of the *Kcnt1* gene encoding Slack (K_Na_1.1) Na^+^-activated K^+^ channels

**DOI:** 10.1038/s41598-020-60028-z

**Published:** 2020-02-21

**Authors:** Imran H. Quraishi, Michael R. Mercier, Heather McClure, Rachael L. Couture, Michael L. Schwartz, Robert Lukowski, Peter Ruth, Leonard K. Kaczmarek

**Affiliations:** 1 Department of Neurology, Yale University School of Medicine, New Haven, CT USA; 2 Department of Neuroscience, Yale University School of Medicine, New Haven, CT USA; 3 Pharmakologie, Toxikologie und Klinische Pharmazie, Institut für Pharmazie, Tübingen, Germany; 4 Departments of Pharmacology and Cellular and Molecular Physiology, Yale University, New Haven, CT USA

**Keywords:** Transgenic organisms, Ion channels in the nervous system, Epilepsy, Experimental models of disease, Epilepsy

## Abstract

Gain-of-function mutations in *KCNT1*, the gene encoding Slack (K_Na_1.1) channels, result in epilepsy of infancy with migrating focal seizures (EIMFS) and several other forms of epilepsy associated with severe intellectual disability. We have generated a mouse model of this condition by replacing the wild type gene with one encoding *Kcnt1*^R455H^, a cytoplasmic C-terminal mutation homologous to a human R474H variant that results in EIMFS. We compared behavior patterns and seizure activity in these mice with those of wild type mice and *Kcnt1*^−/−^ mice. Complete loss of *Kcnt1* produced deficits in open field behavior and motor skill learning. Although their thresholds for electrically and chemically induced seizures were similar to those of wild type animals, *Kcnt1*^−/−^ mice were significantly protected from death after maximum electroshock-induced seizures. In contrast, homozygous *Kcnt1*^R455H/R455H^ mice were embryonic lethal. Video-EEG monitoring of heterozygous *Kcnt1*^+/R455H^ animals revealed persistent interictal spikes, spontaneous seizures and a substantially decreased threshold for pentylenetetrazole-induced seizures. Surprisingly, *Kcnt1*^+/R455H^ mice were not impaired in tasks of exploratory behavior or procedural motor learning. These findings provide an animal model for EIMFS and suggest that Slack channels are required for the development of procedural learning and of pathways that link cortical seizures to other regions required for animal survival.

## Introduction

Sodium influx into neurons through voltage-dependent sodium channels and through glutamate receptors during repetitive firing in neurons leads to the activation of Na^+^-activated K^+^ currents, termed K_Na_ currents^1^. Many K_Na_ currents are mediated by two related potassium channel subunits^2^. Like many ion channels, these two subunits have had multiple names over time, and the names used here are Slack (also called K_Na_1.1 or Slo2.2 and encoded by the *KCNT1* gene) and Slick (K_Na_1.2, Slo2.1, encoded by the *KCNT2* gene)^3,4^. The Slack (Sequence Like A Calcium-activated K^+^ Channel) subunit is widely expressed in the mammalian nervous system^5^. Rapid activation of K_Na_ currents contributes to action potential repolarization and shapes synaptic potentials, while slower activation produces adaptation of firing rates during sustained neuronal stimulation and to afterhyperpolarizations that follow such stimulation^6^, K_Na_ currents also regulate the temporal accuracy of action potentials in response to high-frequency stimulation^7^.

Slack channels resemble K_v_ channels in their transmembrane topology, with six hydrophobic transmembrane domains^8^. The large cytoplasmic C-terminal domain of the Slack subunit, however, shares similarities with the BK potassium channel^9^ and contains two RCK (regulator of the conductance of K) domains. In addition to having a putative Na^+^ sensing domain^10^, the C-terminus is required for the interactions between the Slack protein and cytoplasmic signaling molecules such as Phactr-1 and the Fragile X Mental Retardation Protein (FMRP)^11,12^.

Mutations in *KCNT1*, the gene that encodes Slack channels, have been implicated in a spectrum of genetic focal epilepsies associated with cognitive and behavioral deficits. In particular, mutations have been identified in over half of patients with epilepsy of infancy with migrating partial seizures of infancy (EIMFS), also called malignant migrating partial seizures of infancy (MMPSI)^13–15^, an intractable infantile epilepsy associated with severe intellectual disability. Mutations have also been reported in a series of patients with autosomal dominant nocturnal frontal lobe epilepsy (ANDFLE)^15,16^, a familial epilepsy syndrome with onset in late childhood through early adulthood, as well as in Ohtahara syndrome^17^, another severe early infantile epileptic encephalopathy. Most of these disease-causing mutations result in single amino acid changes within the extended cytoplasmic C-terminal domain of the Slack protein.

Analysis of multiple epilepsy-associated mutations from EIMFS, ADNFLE, and Ohtahara syndrome patients in heterologous expression systems has demonstrated that the mutations produce 3- to 22-fold increases in Slack current^13,15,17,18^. An ~20-fold increase in K_Na_ current has also been found in neurons derived from human IPS cells expressing an EIMFS mutation^19^. Two studies that recorded single channel activity in heterologous expression systems found that some, but not all, of these mutations produce changes in voltage-dependence or sodium-sensitivity that increase open probability^15,20^. These increases are, however, markedly smaller than the increases recorded in macroscopic recordings or in membrane patches containing multiple channels. This finding is consistent with the hypothesis the mutations increase cooperativity among channels within a cluster and is consistent with the fact that most of the mutations are located in the C-terminal cytoplasmic domain that is required for protein-protein interactions of these channels^15^.

To determine whether a heterozygous gain-of-function mutation in the Slack channel is sufficient to cause seizures, we generated a mouse line bearing the R455H mutation in *Kcnt1*, homologous to the human disease-causing variant R474H. In a heterologous expression system, this EIMFS-associated homologous mutation produced an increase in potassium current that was greater than that of several other disease-causing mutations^15^. We have compared seizure thresholds and responses to induced seizures in these mice with wild type animals as well as those of *Kcnt1* knockout mice^21^. In addition, we have characterized behavioral impairments in procedural learning in both sets of animals, in an extension to previous behavioral studies on this particular knockout mouse model^21,22^ and similar models from other groups^23^.

## Results

### Loss of Slack channels increases survival after seizures

We first tested the effects of deletion of the rodent *Kcnt1* gene on seizure activity. As described in the methods these mice were generated on a background of Sv/129 and backcrossed at least twice to C57BL/6N. The wild type controls in these experiments were a parallel colony of Sv/129 backcrossed to C57/BL6N as have been used in other studies of *Kcnt1*^21,22,24^. Two *Kcnt1*^−/−^ and two wild type mice on the same background were implanted with EEG electrodes. No spontaneous seizures were found during 72 continuous hours of recording. No epileptiform discharges were identified using either automatic spike detection software or manual review of the recordings. In addition, no spontaneous seizure-like behaviors were observed in any of the *Kcnt1*^−/−^ or corresponding wild type mice used in this study.

**Figure 1:**
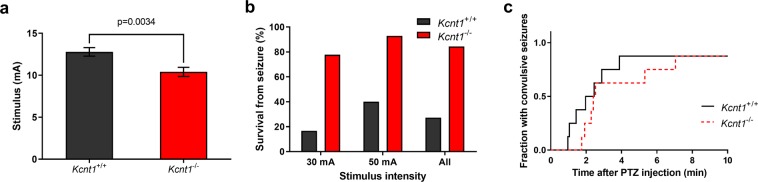
Acute seizure induction in *Kcnt1*^−/−^ mice and age-matched wild type controls. (**a**) Threshold stimulus current to evoke a tonic hindlimb extension seizure. Seizure thresholds are reduced in *Kcnt1*^−/−^ mice compared to wild type (p =< 0.01, Student’s t test). (**b**) Electroshock seizure survival at high stimulus intensities. Seizures induced by these high currents were associated with high mortality in wild type animals, while *Kcnt1*^−/−^ mice were relatively protected (Pearson’s χ^2^ < 0.01). (**c**) Latency to convulsive seizures (generalized clonus, rearing and falling, or uncontrolled jumping) following administration of PTZ 60  mg/kg *i.p*. No differences were observed either in time to seizures (p = 0.27, log rank test) or in observations in the pattern and intensity of seizures.

To determine whether the susceptibility of *Kcnt1*^−/−^ animals to induce seizures is different from that of wild type animals, mice at age 6–8 weeks were subjected to electroshock using a ramp protocol. Hindlimb tonic extension seizures could be elicited in all mice tested. Electroshock seizure thresholds were slightly decreased in *Kcnt1*^−/−^ animals as compared to age-matched wild type controls (10.4 vs. 12.8  mA, p = 0.0034, N = 28, two-tailed t test) (Fig. 1a).

More strikingly, *Kcnt1*^−/−^ mice had a large reduction in mortality from electroshock seizures. This became clear with the induction of seizures at higher currents. Analysis of higher current stimulation was initially done in an attempt to gauge seizure severity by measuring the ratio between flexion and extension phases of tonic hindlimb seizures at current intensities that were high enough to avoid the effects of seizure threshold variability on the results^25^. The ratios could not be calculated accurately because of a high mortality rate during the extension phase in the wild type mice. Surprisingly, the Slack^−/−^ mice were mostly protected from this effect (Fig. 1b). With 30  mA stimulation, the mortality rate associated with loss of Slack was reduced from 83% to 22% (N = 24). This effect persisted at 50  mA where it was reduced from 60% to 7.1% (N = 24). Over all tests, there was a relative risk reduction of 78% (Pearson’s χ^2^ < 0.01).

**Figure 2:**
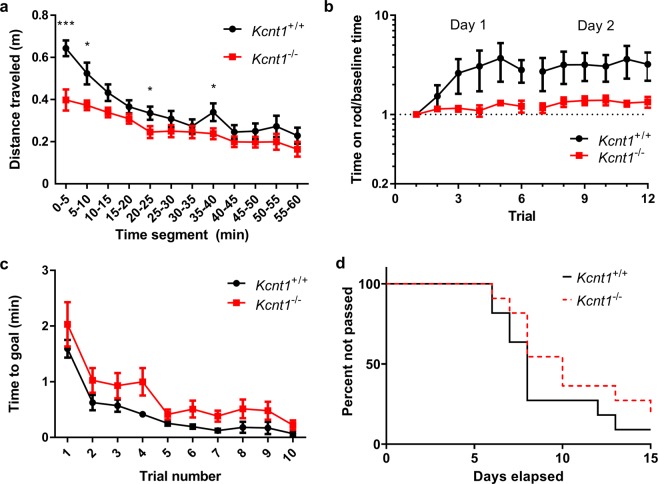
Behavioral screening of *Kcnt1*^−/−^ mice and age-matched wild type controls. (**a**) *Kcnt1*^−/−^ mice had less movement than age-matched controls as demonstrated by a smaller distance traveled (repeated measures ANOVA followed by false discovery rate method, *p < 0.05, **p < 0.01, ***p < 0.001). (**b**) *Kcnt1*^−/−^ mice failed to learn a motor task (accelerating rotarod) over successive trials, unlike corresponding wild type controls which improved over time. Expected final performance scores (*y*_max_) from fit to exponential learning model: 224% (95% CI 136% to 310%) in wild type animals and 40% (95% CI 4% to 77%) in *Kcnt1*^−/−^ mice. (**c**) On a Lashley III maze, a low stress spatial learning task, the time for *Kcnt1*^−/−^ to reach the goal chamber was not clearly different in *Kcnt1*^−/−^ animals than wild type controls (p = 0.10, repeated measures ANOVA), and there was also no difference in how many days it took for *Kcnt1*^−/−^ animals to learn the task successfully (no errors on two successive trials) as compared to wild type (p = 0.22, log rank test).

We also tested the differences between wild type and *Kcnt1*^−/−^ mice in sensitivity to chemically induced seizures by administration of 60  mg/kg pentylenetetrazole (PTZ) at age 6–8 weeks. In all animals, seizures were induced within 10  minutes of administration of PTZ. No differences were found in the latencies to phase 3 seizures^26^ (p = 0.27, N = 14, log rank test), which were 148  s and 132.5  s for Kcnt1^−/−^ and wild type mice respectively (Fig. 2c). At this dose, no mortality was observed for either the wild type or *Kcnt1*^−/−^ mice.

### Kcnt1^−/−^ mice are deficient in procedural learning

We also compared wild type and *Kcnt1*^−/−^ animals in tests of some basic mouse behaviors at age 8–11 weeks. We first confirmed previously reported differences in exploratory behavior^24^ and then extended them to a procedural learning paradigm. In an open field test, which tests a variety of functions related to exploratory behavior including locomotor activity and anxiety, *Kcnt1*^−/−^ mice had overall decreased movement (Fig. 2a, p = 0.016, N = 22, repeated measures ANOVA). The total distance traveled over 60  minutes was 3.15 ± 0.22  m (SEM) in *Kcnt1*^−/−^ animals and 4.22 ± 0.34  m in wild type (p = 0.0079, N = 22, two-tailed t-test). Individual 5-minutes time bins were evaluated individually, and differences were seen in minutes 0–5 (p < 0.001, false discovery method), 5–10 (p = 0.014), 20–25 (p = 0.046), and 35–40 (p = 0.049). Correspondingly, total rest time was greater in *Kcnt1*^−/−^ animals (27.8 ± 1.22  min) than in wild type animals (24.8 ± 1.17  min, p = 0.047, two-tailed t-test), and these differences were also again particularly clear in the first 10  minutes of recording (3.79 ± 0.31  min in *Kcnt1*^−/−^ animals vs. 2.65 ± 0.25  min in wild type mice, p = 0.01).

Motor skill learning was tested by measuring the time animals were able to maintain balance on a rotarod with repeated trials (Fig. 2a). On the first day, animals were placed on an accelerating rotarod for 6 sequential trials, and this procedure was repeated the next day. Wild type animals improved their performance over the trials in the first day, and this improvement was maintained during the second day. This procedural learning was markedly impaired in the *Kcnt1*^−/−^ animals. Baseline performance as measured on the initial trial for each animal was similar between groups. Performance was measured based on the latency to fall on each trial divided by the individual animal’s baseline performance. *Kcnt1*^−/−^ mice had worse performance relative to the wild type group (p < 0.001, N = 22, F test). Maximal expected motor learning performance (improvement over baseline) was 224% (95% CI 136% to 310%) in wild type animals and 40% (95% CI 4% to 77%) in *Kcnt1*^−/−^ mice, a difference that is statistically significant (p = 0.0001, two-tailed t-test).

We also tested spatial learning using a Lashley III maze, which tests the ability of the animals to learn the path to their home cage through a maze that contains four different alleys. Training of wild type and *Kcnt1*^−/−^ animals took place over two days, and the criterion of successful learning was navigation to the home cage with no errors on two successive trials. No differences between wild type and *Kcnt1*^−/−^ mice in this form of spatial learning (Fig. 2c,d). The median number of trials to pass testing was 8 for *Kcnt1*^−/−^ animals and 10 for wild type (p = 0.22, N = 22, log rank test).

### Heterozygous Kcnt1^+/R455H^ mice have spontaneous seizure activity

Another set of mice were generated with the *Kcnt1* R455H missense mutation, which was chosen because this produced the largest gain of function seen in an analysis of multiple epilepsy-associated mutations^15^. As noted in the methods these mice were generated on a C57BL/6J background and backcrossed at least 4–5 generations with wild type C57BL/6J. *Kcnt1*^+/R455H^ animals had essentially normal survival (to over a year) with only one case of unexpected early death (at 2 months) noted among over 200 animals. Although embryos and their brains appeared normal on gross examination, *Kcnt1*^R455H/R455H^ pups produced from heterozygote pairs were invariably stillborn. The mechanism was not clear though respiratory compromise was considered a possibility.

**Figure 3:**
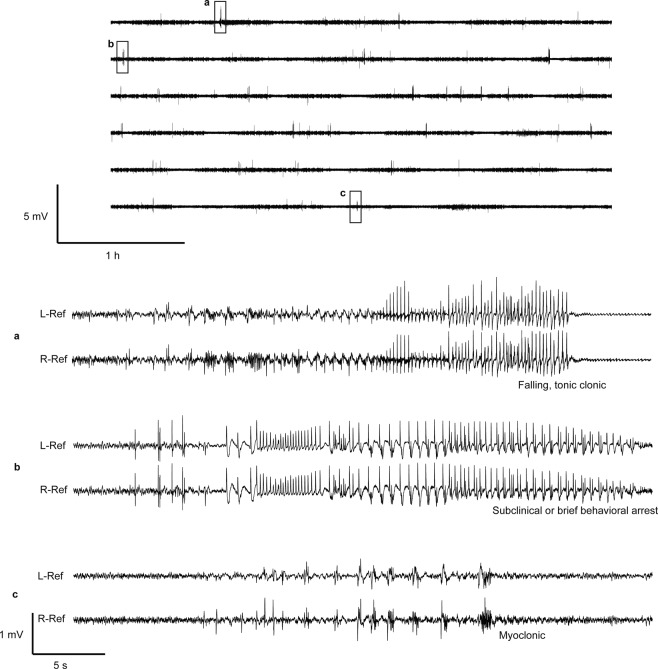
Left hemispheric channel (upper panel) of 24-hour EEG recording from a *Kcnt1*^+/R455H^ heterozygous mouse demonstrating three spontaneous seizures. Multiple seizures types were seen including (**a**) falling with tonic-clonic movements, (**b**) subclinical seizures or possible brief behavioral arrest, and (**c**) myoclonic seizure. No seizures or interictal discharges were seen in the corresponding wild type littermates (N = 4) or in *Kcnt1*^−/−^ animals (N = 4) in 72  hours of recording.

**Figure 4:**
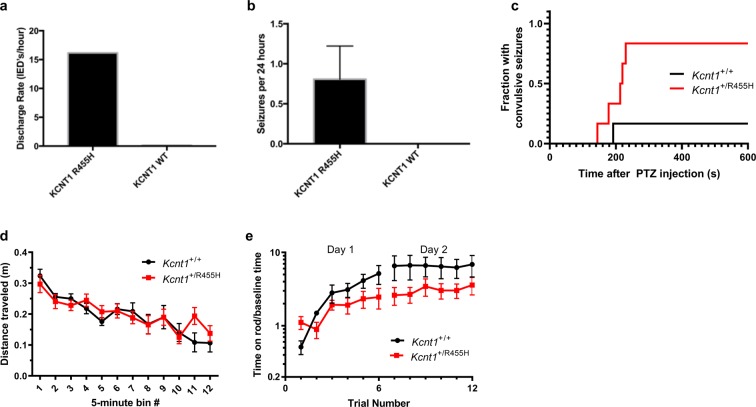
Seizures and behavior in *Kcnt1*^+/R455H^ heterozygous mice. (**a**) All *Kcnt1*^+/R455H^ mice had interictal epileptiform discharges (IEDs) on EEG with a mean of 16 spikes per hour, as compared to no interictal discharges in the wild type littermates. (**b**) The mean spontaneous seizure rate in 72  hours of recording was 0.7 seizures/day in *Kcnt1*^+/R455H^, in contrast to no seizures seen in the wild type group. (**c**) Administration of PTZ 60/mg/kg *i.p*. induced more convulsive seizures in mice carrying the epilepsy-associated variant than in wild type littermates (N = 6 per group) with a significant difference in latency to seizures (p = 0.03, log rank test). (**d**) Open field behavior was similar between *Kcnt1*^+/R455H^ and corresponding wild type mice, as demonstrated by the distance traveled over one hour. There was no difference in movement over the course of the hour (p = 0.92, repeated measures ANOVA). (**e**) There was no significant difference in rotarod performance between *Kcnt1*^+/455H^ and wild type littermates, with performance measure based on 833% (95% CI 102% to 1370%) for wild type littermates and 378% (0% to 836%) for *Kcnt1*^+/R455H^.

*Kcnt1*^+/R455H^ mice and wild type controls at age 8–10 weeks were evaluated with continuous video EEG monitoring for 72  hours (Fig. 3). All *Kcnt1*^+/R455H^ mice that underwent recording had interictal epileptiform discharges (mean 16/hour, N = 7; Fig. 4a). Over this time period, electrographic seizures were identified in 3/7 animals (0.8 seizures/day over all animals; Fig. 4b). No interictal discharges or electrographic seizures were seen in wild type littermates (N = 7).

Following administration of PTZ 60  mg/kg, convulsive (phase 3) seizures^26^ were observed in 5 out of 6 *Kcnt1*^+/R455H^ mice and 1/6 wild type littermates at age 8–10 weeks (Fig. 3c). In *Kcnt1*^+/R455H^ mice, the median latency to a seizure was 216.5  seconds (N = 6). This could not be calculated in wild type mice due to lack of convulsive seizures at this dose. Seizure latencies based on survival analysis were significantly different (p = 0.033, N = 12, log rank test).

### Slack R455H mice have normal procedural learning

Open field performance was measured in *Kcnt1*^+/R455H^ mice (age 8–10 weeks) and was no different from wild type littermates (Fig. 4d). Total distance traveled in 60  minutes was 2.36 ± 0.20  m (N = 7) for wild type and 2.43 ± 0.15  m (N = 9) for *Kcnt1*^+/R455H^ mice (p = 0.79). Individual time bins were scored and compared. There were no differences in movement between genotypes (p = 0.92) or gender (p = 0.75). There was also no difference in rest time between genotypes (p = 0.94) or gender (p = 0.80). Comparisons were done using repeated measures ANOVA.

Motor skill learning was tested with repeated rotarod experiments (age 8–11 weeks) measuring the ability of animals to maintain balance on an elevated rotating rod (Fig. 4e,f). Differences between *Kcnt1*^+/R455H^ and their wild type littermates were not as clear as between *Kcnt1*^−/−^ and their wild type controls, and there was more variability. Performance improvement showed large variability and overlapping confidence intervals. The model-based performance score was 833% (95% CI 102% to 1370%) for wild type and 378% (0% to 836%) for the mutation. Due to the large confidence intervals in the model fits, we also looked directly at the actual scores in the final trial, which were 686 ± 223% (N = 7) for wild type and 359 ± 96% (N = 5) for wild type, and not significantly different (p = 0.21, Student’s t test). Finally we did not find a difference between male and female *Kcnt1*^+/R455H^ mice (p = 0.80, ANOVA, 5 female and 4 male).

## Discussion

Our findings using mice that either lack Slack channels or express one copy of a gain-of-function mutation in this channel are consistent with the hypothesis that an increase in neuronal K_Na_ current results in epilepsy. The results correspond to the behavior of induced pluripotent stem cell-derived neurons, which also showed hyperexcitability^19^. *Kcnt1*^−/−^ animals have no spontaneous seizures and are no different from wild type animals in their sensitivity to seizure induction by PTZ, although there is a mild reduction in the threshold for electroshock-induced seizures. Surprisingly, a major consequence of deletion of *Kcnt1* is strong protection from electroshock seizure-induced mortality. Although the biological basis of this is not yet known, it may indicate alterations in the development of connections between the cortical regions with high Slack protein expression and brainstem areas that regulate breathing. Given the recent emphasis on understanding sudden unexpected death in epilepsy (SUDEP) this finding deserves further exploration^27^. In contrast to wild type and *Kcnt1*^−/−^ animals, heterozygous mice that have a single copy of R455H mutation have persistent interictal spikes and spontaneous seizures, as well as greatly increased sensitivity to PTZ and increased mortality following seizures. Mice that are homozygous for R455H are not viable, although they appear normal *in utero*. In this respect, they match EIMFS patients, in whom the disease is caused by only a single copy of this and other gain-of-function mutations^15^. Only one patient homozygous for mutant *KCNT1* has been reported, in a case of uniparental disomy for A966T^17^; however that is a rarity, and the gain of function of the rodent homolog channel was lower than for R455H^15^.

At the level of single neurons, loss of K^+^ channels typically promotes neuronal hyperexcitability, while increases in K^+^ currents reduce excitability. Consistent with this, *Kcnt1*^−/−^ mice had lowered thresholds for maximal electroshock seizures, a small but statistically significant effect. Although one loss-of-function *KCNT1* mutation has been described^28^, the vast majority of epileptogenic *KCNT1* mutations are gain-of-function mutations that increase K^+^ current^13,15,18^, which has now been confirmed pathogenic by the *Kcnt1*^+/R455H^ mice. There are several potential explanations for how gain of function in Slack current may lead to seizures. First, it is known that increases in K^+^ currents that activate selectively at positive potentials during an action potential increase the ability of neurons to fire at high rates^29^. Although the intrinsic voltage-dependence of Slack channels does not match that of channels that activate selectively at positive potentials, they are activated by Na^+^ ions and maximal Na^+^ influx occurs at positive potentials during the upstroke of an action potential. Thus, if gain-of-function Slack mutant channels activate rapidly in response to Na^+^ influx during an action potential this could promote hyperexcitability. This idea has been supported by iPSC-derived neurons that carry an MMPSI-associated Slack mutation, as they have shorter action potentials and larger afterhyperpolarizations allowing more rapid firing^19^. Second, suppression of firing in inhibitory neurons by increased K^+^ current could trigger seizure activity. Arguing against this possibility is the finding that Slack channels are expressed at higher levels in glutamatergic neurons of the mouse and rat cerebral cortex than in inhibitory interneurons, some of which express little or no *Kcnt1* mRNA^5,30,31^. Third, increased excitability could result from an altered pattern of synaptic connections rather than (or in addition to) a change in intrinsic excitability. This hypothesis is consistent with the finding that seizures do not propagate across the cortex or across the corpus callosum in patients with EIMFS^32^. Definitive resolution of whether one or more of these factors results in epileptogenesis will require substantially more cellular and systems analysis of the Slack^+/R455H^ animals.

Despite the striking effect of the *Kcnt1*^+/R455H^ mutation on cortical excitability, the effects of this mutation on overall behavior appear to be less severe than in *Kcnt1*^−/−^ animals. No significant effects are seen on behavioral screening tests in *Kcnt1*^+/R455H^ mice, while *Kcnt1*^−/−^ mice have markedly reduced exploratory behavior and impaired procedural learning. The finding in *Kcnt1*^−/−^ mice is consistent with studies demonstrating these mice have normal working memory and control of motor functions but are impaired in tasks that require cognitive flexibility^21^. The decreased open field movement^24^ and impairments in motor learning in *Kcnt1*^−/−^ mice are suggestive of an anxiety-like or autistic phenotype with absence of Slack function. The motor learning changes are the most evident. Motor coordination on rotarod testing was previously measured as unimpaired in *Kcnt1*^−/−^ mice using a paradigm that did not assess learning^21^. This finding is similar to the present results that we observed on the first rotarod trials. With subsequent trials, however, we found that the typical pattern of saturable improvement in performance in wild type animals was completely absent in *Kcnt1*^−/−^ mice. These impairments may be related to impaired plasticity due to loss of the Slack-dependent interactions with cytoplasmic cell signaling pathways, such as those that influence neuronal protein translation and plasticity^33^. Nevertheless, we found no differences in spatial learning between wild type and *Kcnt1*^−/−^ mice using a Lashley III maze, which likely requires neural mechanisms quite distinct from those required for procedural motor learning.

The discrepancy between abnormal behavior seen in *Kcnt1*^−/−^ mice and epilepsy seen in *Kcnt1*^+/R455H^ mice suggests that these two phenotypes involve different pathways. The behavioral phenotype in *Kcnt1*^−/−^ mice may have to do with known protein-protein interactions between the channel and cellular cytoplasmic signaling pathways involved in learning and development. The cytoplasmic C-terminus of the Slack protein binds the Fragile X Mental Retardation Protein (FMRP). Absence of FMRP leads to Fragile X syndrome, which is the most common cause of inherited intellectual disability and is also associated with an enhanced occurrence of childhood seizures^34^. FMRP regulates neuronal protein synthesis by binding to ribosome-mRNA complexes containing RNAs encoding proteins known to be required for normal neuronal plasticity and development^35^. The binding of FMRP to Slack increases its open probability^13,33^. As *Kcnt1*^+/R455H^ mice have one wild type copy of the gene this may be sufficient to avoid the behavioral consequences in the mouse model. Unlike procedural learning and open field behavior, spatial learning in *Kcnt1*^−/−^ animals was not impaired and suggests that Slack function may be less critical in the hippocampus than in other brain regions (correspondingly, the highest cerebral expression is in the frontal cortex). This hypothesis is consistent with the neocortical (frontal lobe and migrating multifocal) seizures seen in patients with *KCNT1* mutations^13,15,16,36^.

Human diseases associated with Slack channel gain of function do have a significant cognitive component^34^. The mouse R455H *Kcnt1* mutation corresponds to the human mutation, *KCNT1* R474H, which causes very early onset epilepsy, together with very severe intellectual disability. The intellectual dysfunction could be due to the frequent seizures, to aberrant development from abnormal firing patterns, or due to effects on Slack’s protein-protein interactions described above. Interestingly, *KCNT1* gain-of-function mutations also give rise to other epilepsies including ANDFLE^15,16^, which has an onset later in childhood, typically at ~8–10 years. ADNFLE can also be caused by nicotinic acetylcholine receptor mutations, which produce a very similar pattern of frontal lobe epilepsy but are not typically associated with the very severe psychiatric and cognitive symptoms produced by Slack mutations. The difference between the two suggests that the human mutations have cellular effects beyond the simple control of excitability.

This initial study has a number of limitations, some of which need to be addressed in future work. The epileptic and behavioral phenotypes were only tested in adult animals, whereas the human disease presents in infancy, so evaluation of early ages will be important. The knock-out and knock-in mice are on somewhat different background strains, and were compared to their separate wild types, but were not able to be compared to each other directly. The seizure induction tests, although they used generally established or known methods, did not precisely follow those of the NINDS antiepileptic drug screening program, which could be relevant in the future if these mice are used for a drug screening endeavor.

There are currently no effective treatments for EIMFS or the other conditions produced by gain-of-function *KCNT1* mutations. There have been efforts to treat these epilepsy syndromes with quinidine^18,37^, a non-specific channel blocker that acts on Slack channels^38,39^. This has, however, not proved to be generally effective^40^, likely because of the very large number of other targets affected by quinidine, including those involved in cardiac conduction. The present work demonstrating that *Kcnt1*^+/R455H^ knock-in mice phenocopy many aspects of the human epilepsy provides an animal model for the development of new therapies for these devastating diseases.

## Methods

### Kcnt1^−/−^ mice

*Kcnt1*^−/−^ mice were obtained that were initially generated on a Sv/129 background by homologous recombination in murine embryonic stem cells as previously detailed^21^. *Kcnt1*^−/−^ mice and wild type controls were subsequently backcrossed 2 generations with a C57BL/6 N background strain prior to testing. Backcrossing was confirmed by PCR of *Disc1* as *Disc1* deletions are present in 129 mice. A breeding colony was established at the Yale University Animal Resources Center. Mice were kept on a 12-hour light/dark cycle. Food and water were freely available except as noted. All experiments on this strain were performed with 2 to 3-month-old males weighing 25–35  g. Wild type animals had an identical genetic background as the knockout animals. Genotyping was performed routinely by PCR on genomic DNA isolated from tail-tip biopsies. All experiments were done in accordance with the Yale University Institutional Animal Care and Use Committee. Experimental protocols including invasive EEG recording and seizure induction were approved by this committee (Yale IACUC #2019-07842).

### Kcnt1 R455H mice

The *KCNT1* R474H variant has been associated with early infantile epileptic encephalopathy. The homologous rodent mutation is R455H in exon 15. This variant was chosen because it had the largest electrophysiological change out of multiple epilepsy-associated mutations^15^. The arginine residue here is encoded by CGA in the mouse and rat genes and CAA in the human gene. In the human mutation, histidine is represented by CAC. We aimed for a two nucleotide change from CGA to CAC, because it was the same codon as the human mutation, and because CAC codon usage in the mouse genome is 50% higher than the alternative codon CAT.

The mutation was engineered by homology-directed repair with CRISPR/Cas9^41^ via the Yale Genome Editing Center. The guide sequence CCAGACCATCCTTCGAGCCTGGG was selected because it had no likely off target sites on chromosome 2 and center location within 1 nucleotide of the mutation site (underlined). A dsDNA primer was sequenced containing T7 promoter, the guide sequence (underlined), and a single guide RNA scaffold, as follows: TGTAATACGACTCACTATAGGCCAGACCATCCTTCGAGCCTGTTTTAGAGCTAGAAATAGC. The 160 b.p. ssDNA donor oligonucleotide template with the desired mutation (underlined) was CCGTGCTGCCTGCCTACTCTTAGCAGTAGGCCCTCTAACACCAACCACACCTCCCCACAGGATCACCAGACCATCCTTCACGCCTGGGCTGTGAAGGACTTTGCCCCCAACTGTCCCCTCTATGTCCAGATCCTCAAGCCCGAAAACAAGTTTCACGTCA.

Cas9 mRNA, sgRNA, and donor template were injected into C57BL/6J zygotes that were subsequently implanted into surrogate mothers. Following two rounds of injections, there were a total of 17 surviving pups, of which three had incorrect mutations at the target site, one appeared mosaic for the desired mutation, and one was confirmed heterozygous for R455H and used for subsequent breeding.

A breeding colony was established in the same fashion as for the *Kcnt1*^−/−^ mice. Litters were genotyped by PCR of the region containing the missense mutation followed by Taq1 restriction digest. Although the likelihood of introducing an off-target mutation was predicted to be quite low, mice were further backcrossed another 4–5 generations with wild type C57BL/6J mice for additional reassurance and to breed out any potential off target mutations that might have been missed.

### Electroencephalography

Mice were implanted with 4 epidural screw electrodes (left frontal, right frontal, and posterior reference and ground) to gauge epileptiform activity. After 24  hours or more or recovery, mice were placed in a clear Plexiglas cage, where video EEG recording was performed for 72  hours. Signals were recorded with a CEEGraph EEG system (Biologic).

### Acute seizure induction

Animals were subjected to a 0.2 s-duration sinusoidal constant alternating current at 60  Hz via moistened auricular electrodes through a rodent shocker capable of stimulation up to 750  V (Harvard Apparatus #730105). To measure the threshold of electroshock-induced seizures, stimulation was given via a ramp protocol in 1  mA steps from 0, delivered 2–3  minutes apart, until maximal tonic hindlimb extension was observed. The protocol has previously been used in epilepsy mouse models^25^ and is similar to one originally proposed in a rat model^42^ as an alternative to usual maximum electroshock methods. Differences in thresholds were evaluated by a two-tailed t test with significance at p = 0.05. Mice that had not undergone any experiments in at least 24  hours were given stimuli of 30 or 50  mA to measure the severity of seizures at constant currents high enough to avoid the effects of variable seizure thresholds. Differences in survival were assessed by Pearson’s chi-squared test with significance at χ < 0.05.

Seizures were also induced chemically with pentylenetetrazole (PTZ). Animals that had not undergone any previous seizure induction were given PTZ 60  mg/kg *i.p*. and observed for at least 10  minutes for any signs of seizures^43^. Seizures were timed and graded using a scale adapted from one previously described for PTZ in mice^26^ as follows: phase 0: no seizure; phase 1: hypoactivity or behavioral arrest; phase 2: myoclonus or partial clonus; phase 3: generalized clonus, rearing and falling, or uncontrolled jumping; phase 4: generalized tonic-clonic/tonic hindlimb extension. Survival analysis was performed on the latency after injection for mice to reach phase 3 seizures, and comparison of these curves between groups was done via a log rank test from which a p value was derived with significance reported at a level of 0.05.

### Behavioral testing

Open-field activity was tested by acclimating the mice for at least 30  minutes then placing them in a mouse activity monitoring cage for 60  minutes. Floor plane sensor measurements were collected using TruScan software (Coulburn Instruments) for three sessions on consecutive days. Automated measurements included time moving, distance moved, rest time, and center vs. margin time. Analysis of open field activity (time moved or at rest per per time bin) was performed using repeated measures ANOVA. Sources of variation were time bin, genotype, and a time x genotype interaction term. In cases where multiple genders were tested, the sex of the animals is included as an additional term and reported as well. The significance of the genotype term is reported with significance level of 0.05, and when significant is followed by post-hoc comparison of individual time bins corrected for multiple comparisons using a false discovery rate approach^44^ with Q = 1%.

A rotarod task was used to assess locomotor coordination and learning of motor skills. Mice were habituated to the testing room for at least 30  minutes and then were tested on an accelerating rotarod (Omnitech Electronics) for six subsequent sessions on two consecutive days^45^. A learning score was created in which the time on the rod was divided by the baseline performance for each mouse. For motor learning we expected to see an exponential rise in performance followed by saturation, so the improvement over subsequent trials was fit via least squares regression to a basic two-parameter learning model that captures those features, $y(t)={y}_{{\rm{\max }}}(1-{e}^{-k(t-1)})$ where *y* is the performance on each subsequent trial, *t* is the trial number, and parameters ${y}_{{\rm{\max }}}$ and *k* indicate the maximum expected performance improvement and the learning rate, respectively. The parameter ${y}_{{\rm{\max }}}$is reported as the performance score along with 95% confidence intervals. Learning differences were considered significant for non-overlapping confidence intervals of this parameter. In cases where multiple genders were tested, we additionally performed ANOVA with terms for the genotype and sex of the animals; the contribution of sex is reported as a p value with a 0.05 significance level.

Spatial learning was assessed with a Lashley III maze, which is designed to assess learning and memory in mice without aversive motivators such as food or water restrictions or stressors such as swimming in other tasks^46^. The test was performed once a day for up to 15 days per animal. Each mouse was placed in the start box and allowed to traverse the maze until it reached the goal (home cage). Errors were scored based on deviations from the optimal path or crossing a door incorrectly. An animal was considered to have reached criterion performance if the total errors on 2 consecutive days of testing had been 1 or less. Failure to reach criterion within the 15 days of testing was considered a failure to learn and testing was stopped and trained over two days. Performance between genotypes was compared using a survival analysis of the time to reach the criterion with a log rank statistic.
